# The Institutional Worker

**Published:** 1913-03-15

**Authors:** 


### INSTITUTIONAL WORKER

**Being a Special Supplement to "The Hospital"**

### OUR BUREAU OF INFORMATION.

### Rules for Correspondents.

1. Every letter, on whatever subject, must be accompanied by the coupon to be cut from the back cover (inside page) of The Hospital, current issue, and must contain the name and address of the correspondent with pseudonym for publication if desired.
2. Replies by post cannot be given, save under exceptional circumstances at the Editor's discretion.
3. Letters from Approved Homes in reply to special needs published in the Bureau should state terms and full particulars, and be sent propaid under cover to the Editor of the Bureau with name written across coupon for identification.
4. Proprietors of Homes which have not yet been entered on the List of Approved Homes, but have spare accommodation likely to suit special needs, are invited to write for an application form for registration. The fee for registration, which includes two announcements of the Home in the Bureau and other privileges, is 5s.
5. All communications to be addressed to the Editor of The Hospital, 28 Southampton Street, Strand, London, W.C., and marked "Bureau of Information."

### Invitation to Readers.

**The Editor is prepared to forward to applicants, without fee on either side, offers of accommodation from Homes on the Approved List suitable for medical and surgical cases, for the aged, for chronic or mental patients, for electrical treatment and massage, for convalescents and for children, at varying terms, in any required locality. Reduced terms can often be arranged. The special needs of any who may be sick or in difficulties are also made known in these columns free of charge.**

### Night Nurses' Food.

We recommend you to procure sufficient fireproof earthenware pots, with covers, to go round for each night nurse. The food can be kept hot for hours in these handy receptacles, and a great variety of dishes can be prepared in them. One of the simplest is made by putting smallish pieces of beef or mutton to fill about a quarter of the pot. Season to taste. Cover with thin slices of onion, and fill up the pot with sliced potatoes, sprinkling flour and salt over these. Pour in half a cup of water or stock, put on the lid, and bake in a moderately hot oven, allowing plenty of time. Cold meat may be used if underdone.—"Careful Matron."

### Salt Beef.

We do not consider this a suitable dish under any circumstances for women whose associations call them back to strenuous work directly their meal is over. Even if very lightly pickled, the effect is to render the meat less easy of digestion.—Prudence.

### Electric Light in Lavatories.

We have no faith in pinning up drastic threats. There is no sure way of preventing the absent-minded from leaving the light on, except the simple expedient of an attachment which automatically turns up the light only when the door is bolted. These are easily procured from any good fitter, and should be detachable during the day-time. They are very useful also for telephone boxes, and will speedily save you far more than the initial cost.—Secretary.

### Feeble-minded Fallen Women.

It is not possible to give the exact statistics you require in this connection. It has been estimated by persons who have investigated the subject with some care that about 50 per cent. of the fallen women are to some extent defective. A vast proportion, however, do not come under observation, and this can only be regarded as guess-work. So far as can be ascertained—and this, again, is a very rough computation—the proportion of the feeble-minded to the whole population is 1 in 269.—Observer.

### Vaccination Objectors.

You are right in believing that the number of persons who apply for certificates of exemption from vaccination for their children is increasing all over the country, and this seems to be specially the case in London. In 1911-12 the objections in Lambeth alone totalled 475, as against thirty in 1904-5. The Local Government Board Report shows that the exemptions had risen in 1910 to 233, 677 out of 897,273 births.—G. F. T.

### Home for Defective Child.

A home is wanted for feeble-minded boy of five. He can walk a little, has no disfigurement, nor any objectionable appearance. Seems to be cheerful and good-tempered. Belongs to working-class family who can pay 10s. 6d. a week inclusive, and would prefer him to be with a nurse who understands mental deficiency.—Defective (156a).

### Rescue Homes in Cornwall.

Procure a copy of their Guide (price 6d.) from the Church Penitentiary Association, Church House, Dean's Yard, Westminster, which will give you full information on rescue work in this country. The Home most likely to suit your case is the Epiphany Laundry Home. Payment, £5 a year, or free. There are twenty inmates between the ages of fourteen and twenty.—Visitor.

### Necessitous Out-Patient.

Write to the Sister-in-charge, the House of Charity, Greek Street, Soho, which is quite free as a temporary residence for respectable persons of all grades of society. They frequently accommodate out-patients who have come up for treatment at any of the London hospitals, but cannot afford to live at their own expense. There are no formalities, and your own letter of recommendation, explaining the circumstances, together with one from the vicar of the parish, will be sufficient.—Miss Sarah H.

### Preliminary Training at Guy's Hospital.

The course lasts six weeks, and the fee charged (which covers the entire expense) is six

### Tuberculosis in Birmingham.

Your query relating to the conditions which have made Birmingham a hot-bed of consumption can best be answered by the age statistics which have been collected by the Birmingham Health Committee. From these it would appear that bad industrial conditions, unhealthy factories and workshops, are largely more to blame than the sanitary defects; the town is healthily placed, and its municipal work is efficiently carried out. The death-roll annually from this cause is 1,200, while 10,000 persons are estimated to be affected. Of those who die, the number under fifteen is 1 per cent.; while the number who die between fifteen and twenty-five—that is, after regular work in factories has commenced—rises to 36 per cent. It is clear that in many trades the mortality from consumption calls for stringent regulations.—K. M. P.

### Homes for the Aged Poor.

The only Poor-Law Homes of this nature of which we have any knowledge are those at Leeds, Sheffield, Bradford, Dewsbury, and Hull. No doubt you could get particulars of the cost and obtain permission to view from the Clerk of the Guardians in any of these towns. At Bristol a proposal is under consideration for boarding out the aged in specially selected families. We foresee difficulties in carrying out this scheme, but it has many tempting features.—Reformer.

### Maternity Benefit.

The newly formed National Association for the Welfare of Infancy, of which the King and Queen have become patrons, while Mr. John Burns is president and Sir Thomas Barlow chairman, is occupied with the difficulties to which you refer. In many localities committees of women are being formed to take a personal and effective interest in every case, and provide as far as possible in doubtful circumstances for securing the benefit to the mother's sole use.—Mary R.

### Diet for Fever Convalescents.

*Breakfast.* — Tea or cocoa, bread and butter or dripping, bacon once a week for adults, jam and marmalade.

*Tea.*—Bread and butter, jellies for delicate patients, and always jam or marmalade.

*Supper.*—Children : Bread and milk or bread and butter and cocoa. Adults : Tomato soup, potted ham or beef, fish cakes or porridge, stewed fruit when in season, and occasional junket or custard.

*Dinners.*—Adults : A chop or steak on Sunday, with Yorkshire pudding, potatoes, and a boiled suet pudding, sometimes plain with syrup, or sometimes made with currants and sultanas. Children : Sunday.—Stewed beef and rabbit, with carrots, onions, and mashed potatoes, and milk puddings (both rice and sago). Monday and Friday.—Stewed neck and breast of mutton, with onions and vegetables, mashed potatoes, and milk puddings. Tuesday and Thursday.—Fish, fried for adults, with melted butter sauce and fish cooked in the oven with water, salt and dripping for the children, mashed potatoes, and milk puddings. Wednesday and Saturday.—Either minced beef or beef and potato hash, with puddings. About three days a week adult patients have either a bread pudding with currants (baked) or a boiled suet pudding, and in case of men who have lost much flesh a baked Yorkshire pudding daily. My average expenditure per head, including staff and patients, for thirteen weeks worked out at 6/3 per week.—NORTH-COUNTRY FEVER.

### Widow Left Destitute.

APPLY at once to the Society for the Relief of Distressed Widows, 32 Sackville Street, London, W. Relief is given on subscriber's recommendation within one month of the husband's death. As there are no children this will give opportunity for finding employment and will tide over the worst time.—HOPE.

### Laundry for Sanatorium.

WE understand that at present your washing for a sanatorium with twenty-nine patients and a staff of ten all told costs 25s., not reckoning the food of one laundress by the day. You wish to know whether it would not be more economical as well as more satisfactory to have a small laundry added to the sanatorium, procure hand machinery, and employ a resident laundress. We are fortunately able to supply you below with the figures for laundry in a sanatorium of very similar size to your own, where, however, the machinery is worked by steam. You will notice that two laundry-maids are required, though the numbers are thirty-two as against your thirty-nine. When several maids are employed it is usual to reckon that each one can get through 1,000 pieces a week, but we doubt if a single-handed laundry-maid could get through this amount. You are quite right in estimating that it would be true economy to purchase some labour-saving appliances. We will give you a list in our next issue of some useful hand-worked apparatus for a small establishment. The cost of erecting a small annexe on the sanatorium grounds for the purpose would be trifling, and there can be no doubt at all about the desirability, irrespective of economy, of having the clothes of the particular class of patients you receive washed on the premises.—A. D., Matron.

guineas. The pupils receive preliminary tuition in anatomy, physiology, hygiene, dispensing, bandaging, the making of dressings, use of instruments, bed-making, house-work, sick-room cookery, and other subjects. There are fifteen pupils, who are boarded and lodged in a separate department of the Henriette Raphael Nursing Home. Apply to the Matron, and be prepared to wait for a vacancy. Applicants must not be under twenty-three years of age.—LETTICE.

### Lip Reading.

APPLY to the Association for the Oral Instruction of the Deaf and Dumb, 11 Fitzroy Square, London, W. There is no reason why you should not acquire facility, although you are no longer young. Even when the deaf do not see quite clearly what word has been formed on the lips of those speaking, it has been proved that the system aids them by completing their impression of what they have imperfectly heard.—SURDA.

### Russian Nurses.

NURSING is in rather a deplorable state in Moscow. The nurses are not trained and are very often quite useless, as they refuse to do any hard or dirty work. The usual fees are about two roubles a day, between 4s. and 5s. It is necessary to exercise the greatest caution in accepting offers of employment in Russia, and we recommend you on no account to accept the offer of an agent who is known to you merely through an advertisement. H.M. Consul at Moscow (British Consulate) would advise you whether it is a genuine and safe engagement.—S. S.

* * * A New Edition of the Leaflet relating to THE HOSPITAL Bureau of Information is now ready and will be forwarded free of charge on application.

### Institution Facts and Figures.

### Questions for March.

1. GIVE the average cost per head per week for laundry expenses in your institution (*a*) for patients, (*b*) for staff. Mention the number of beds, whether acute medical and surgical cases are received, and whether the washing is done on the premises or by contract outside.

2. Taking any week in the winter months, state what weight of meat was consumed in the institution, less bacon and ham. Divide this amount by the total number of inmates on meat diet, and thus show the average weight of butcher's meat consumed per head per week. In estimating the numbers include both staff and patients, deducting only those on milk or fish, whose diet does not include beef-tea.

The figures must be the result of actual observation and computation, not estimates. Either or both of the questions may be answered by each contributor. The best answers will be published, and for each contribution considered by the Editor to be of sufficient interest for publication payment of FIVE SHILLINGS will be made.

RULES.

The following rules must be observed by all those answering the Housekeeping Questions :—

1. Answers must be written on one side of the paper. They must bear the name and address of the sender and be accompanied by coupon to be cut from the back cover (inside page) of the current issue of THE HOSPITAL. A pseudonym must be chosen if the name is not to be published.

2. Answers must be addressed to the Editor of THE HOSPITAL, 28-29 Southampton Street, Strand, London, W.C.; they must be marked in the left-hand corner "Facts and Figures," and must be received before the end of the month.

### No Nurses in Bolivia.

THE foreign colony in the capital is too small to furnish employment, even to one trained nurse; and though it is believed on the spot that a demand exists, it is hopeless to attempt to develop it at present. Even medical attendance is sometimes unobtainable, as only doctors with a Bolivian diploma are allowed to practise. Sick nursing is done by untrained nurses.—K. G.

### Fees in Madrid.

NURSING fees are about the same as in England. There is a British Nursing Association, Calle Ayala 15, Madrid, to the superintendent of which you should address your inquiry. The demand for English nurses is limited, but increases. Knowledge of Spanish is essential, as you would probably have to work under doctors of this nationality.—C. E. G.

### The Editor's Letter-Box.

### Communications have been received and attended to from:—

V. D. P.—Yes; we did mention your name in replying to our correspondent.

H. J. W., Redhill.—We regret we are unable to help you. We do not undertake to recommend private nurses, and must refer you to our advertisement columns.

J. W. B.—We gladly welcome your co-operation, and will insert your suggested queries shortly. The daily diet sheet has many points of interest.

H. M. B.—Your laundry figures are excellent.

### Editor's Notices.

**Contributions:**

Contributions should be written, or preferably typed, on one side of the paper only, and all articles sent in are accepted upon the distinct understanding that they are forwarded to THE HOSPITAL only.

The Editor cannot undertake to return MSS. not used, but when a stamped directed envelope is enclosed the MSS. may be returned if a special request is made.

Accepted articles and paragraphs of news will be paid for after publication at the scale rate.

**Address:**

To prevent delay all contributions and letters on editorial business must be addressed exclusively to the Editor, "The Hospital," 29 Southampton Street, Strand, London, W.C. It is important that this regulation shall be strictly observed.

**Correspondence:**

Correspondence on all subjects is invited. The name and address of correspondents must be given as a guarantee of good faith, but not necessarily for publication.

**Special Articles:**

Special articles are invited, and questions, inquiries, and paragraphs upon all matters relating to the work, administration, and management of general, special, mental, fever, cottage and convalescent hospitals, sanatoria, homes, institutions, societies and organisations for the treatment or care of the sick, injured and dependants of all classes. Every matter affecting the interests and welfare of the staff of all grades working in these institutions will receive special consideration.

**Administrative Medicine, Research and Items of News:**

Special terms will be paid for approved articles on subjects in this wide field by experts who have made some section of it their special study and interest. We wish to give prominence to every movement tending to advance accuracy of diagnosis, efficiency in treatment, and the development of modern methods to eradicate disease and promote the welfare of the suffering.

**A Bureau of Information:**

We invite inquiries and applications for information and help in providing for that numerous class of sufferers who require special care or house-room which they cannot obtain in their own homes or secure for themselves. **We cannot, however, prescribe, or recommend practitioners.**

**Books for Review:**

Publishers are particularly requested to send advance proofs of any new books of importance whenever possible, as the Editor has made arrangements to publish immediate reviews on a new plan.

**Photographs, Plans, Blocks, and Illustrations:**

It is requested that wherever possible MSS. may be accompanied by illustrations in any of the above forms. The name of the sender and of the article to which it belongs should be written on the back of each photograph, drawing, or block for purposes of identification.

**Local Papers:**

Newspapers containing reports or news paragraphs should be marked and addressed to the Sub-Editor.

**The Coupon System:**

A coupon will be found at the bottom of the third inside page of the cover each week. This coupon must be attached to every question or inquiry to which an answer is desired in THE HOSPITAL.

### Essential to Institution Workers.

THERE is no Journal other than THE HOSPITAL that so comprehensively covers the whole Institutional World. It is essential not only to those responsible for the Construction, Administration, and Maintenance of Institutions generally, but to every individual worker in any department of the Medical, Health, and Sanitary Services. Useful and authoritative articles on Institution, Poor-Law, and Municipal Affairs, as well as the all-important question of National Insurance, are contained within its columns. To keep abreast with the progress in all branches of Institutional Work, it is necessary to secure a copy of THE HOSPITAL every week; to miss even one Number is an irreparable loss, which no keen worker can afford. In these circumstances, you should place a permanent order with your Newsagents, so as to ensure that you obtain a copy of THE HOSPITAL every Thursday, or a subscription may be forwarded to the Publishers at the undermentioned rates.

**Subscriptions** may begin at any time, and are payable in advance. Cheques and Post-Office Orders should be crossed London County and Westminster Bank, Covent Garden Branch, and made payable to the Manager of THE HOSPITAL.

RATES OF SUBSCRIPTION (payable in advance).

| | | |
|---|---|---|
| **UNITED KINGDOM.** | | |
| Three Months (including Postage) | ... ... ... ... | 2s. 0d. |
| Six Months do. | ... ... ... ... | 4s. 0d. |
| Twelve Months do. | ... ... ... ... | 6s. 6d. |
| **FOREIGN AND COLONIAL.** | | |
| Three Months (including Postage) | ... ... ... ... | 2s. 6d. |
| Six Months do. | ... ... ... ... | 5s. 0d. |
| Twelve Months do. | ... ... ... ... | 8s. 8d. |

*SUBSCRIPTION FORM.*

*Please send me* THE HOSPITAL *for*..............*months, commencing with your issue of*.............................., *for which please find enclosed*...........................

*Name*...............................................

*Address* ..........................................

..........................................

*Date*..................................

*To the Manager,*

*"The Hospital" Building,*

*28 & 29 Southampton Street,*

*Strand, London, W.C.*

### Manager's Notices.

**Letters relating to the publication, sale, and advertising departments of "The Hospital" must be addressed to The Manager, "The Hospital,' The Hospital Building, 28 and 29 Southampton Street, London, W.C.**

**Advertisements:**

To ensure insertion, all advertisements for THE HOSPITAL must reach the Manager not later than Wednesday morning in each week. For scale of charges see pages v and 4.

Special Rates will be quoted for those institutions that agree to send all their vacancy, contract, and public notices to THE HOSPITAL each year, so as to make it a file of such announcements for ready reference.

**Remittances:**

**It is especially requested that remittances be made by Cheque or Postal Order, and in halfpenny stamps only when the amount is under sixpence.**

